# Risk assessment does not explain high prevalence of gestational diabetes mellitus in a large group of Sardinian women

**DOI:** 10.1186/1477-7827-6-26

**Published:** 2008-07-02

**Authors:** Cinzia Murgia, Rachele Berria, Luigi Minerba, Simonetta Sulis, Michela Murenu, Elaine Portoghese, Nicoletta Garau, Pierina Zedda, Gian Benedetto Melis

**Affiliations:** 1 Dipartimento Chirurgico Materno Infantile e di Scienza delle Immagini, Sezione di Clinica Ginecologica, Ostetrica e Fisiopatologia della Riproduzione Umana, Universita' degli Studi di Cagliari, Italy; 2 Department of Obstetrics and Gynecology, Case Western Reserve University, 44109, Cleveland, Ohio, USA; 3 Dipartimento di Sanità Pubblica, Universita' degli Studi di Cagliari, Italy

## Abstract

**Background:**

A very high prevalence (22.3%) of gestational diabetes mellitus (GDM) was recently reported following our study on a large group of Sardinian women. In order to explain such a high prevalence we sought to characterise our obstetric population through the analysis of risk factors and their association with the development of GDM.

**Methods:**

The prevalence of risk factors and their association with the development of GDM were evaluated in 1103 pregnancies (247 GDM and 856 control women). The association of risk factors with GDM was calculated according to logistic regression. Sensitivity and specificity of risk assessment strategy were also calculated.

**Results:**

None of the risk factors evaluated showed an elevated frequency in our population. The high risk patients were 231 (20.9%). Factors with a stronger association with GDM development were obesity (OR 3.7, 95% CI 2.08–6.8), prior GDM (OR 3.1, 95% CI 1.69–5.69), and family history of Type 2 diabetes (OR 2.6, 95% CI 1.81–3.86). Only patients over 35 years of age were more represented in the GDM group (38.2% vs 22.6% in the non-GDM cases, *P *< 0.001). Type 2 diabetes in second-degree relatives was equally represented in GDM and non-GDM subjects, while prior poor obstetrical outcomes mostly characterized non-GDM women (17.5% vs 10.6%, *P *< 0.001). The "average risk" assessment better characterized non-GDM patients (76.8% vs 57.8%, P < 0.001). The logistic regression analysis confirmed that Type 2 diabetes in second-degree relatives, prior poor obstetrical outcomes and the "average risk" definition did not predict the development of GDM.

**Conclusion:**

Such a high prevalence of GDM in our population does not seem to be related to the abnormal presence of some known risk factors, and appears in contrast with the prevalence of Type 2 diabetes in Sardinia. Further studies are needed to explain the cause such a high prevalence of GDM in Sardinia. The "average risk" definition is not adequate to predict GDM in our population.

## Background

Gestational diabetes mellitus (GDM) is defined as a "carbohydrate intolerance of variable severity with onset or first recognition during pregnancy"[1].

The prevalence of GDM may range from 1 to 14% of all pregnancies, depending on the population studied, the diagnostic test employed, its glycemic cut-off and the recommendations applied, and it mirrors that of Type 2 diabetes mellitus [2-4]. Important risk factors include higher maternal age, marked obesity (BMI ≥ 30 kg/m^2^), personal history of GDM, ethnicity and family history of Type 2 diabetes in first-degree relatives [5]. The prevalence of GDM increases with age, becoming more frequent over the age of 30 [6,7]. However, a consistent proportion of women with gestational diabetes are under 30 [7-9]. For these reasons both the American Diabetes Association (ADA) and the American College of Obstetrics and Gynaecologist (ACOG) now consider age ≥ 25 years as a risk factor. As for Type 2 diabetes, obesity is also a major risk factor for GDM, and a Body Mass Index (BMI) > 30 kg/m^2 ^classifies a pregnant woman as high risk for GDM [10]. Overweight (BMI between 25 and 30 kg/m^2^) is also reported to be associated with GDM [9] and for a woman to be defined as low risk for GDM by the ADA and the ACOG [10,11] her weight must be considered normal before pregnancy. Ethnicity influences both the prevalence of GDM and the age it occurs [6,12,13] and Type 2 diabetes mellitus [14]. Reported high risk populations are Hispanic, African, Native American, South or East Asian, or those with Pacific Island ancestry. Women with GDM who have a first degree relative with Type 2 diabetes but not Type 1 diabetes have been reported to have an increased risk of GDM, confirming that GDM and Type 2 share the same genetic background [9,15-17]. However, the use of traditional risk factors to identify GDM excludes approximately half the women with GDM [7]. Furthermore, specific risk factors and their degree of influence on GDM prevalence in a specific population are difficult to quantify, and different studies on various populations have shown differing results [6,18]. It is therefore proposed that the definition of high and low risk for GDM of each ethnic group be based on the prevalence of Type 2 diabetes [11]. The use of historic risk factors is now recommended to identify women who should be submitted to diagnostics test for GDM detection. The ADA recommends risk assessment for GDM at the first prenatal visit and the definition of high, average and low risk be made on the basis of presence/absence of risk factors. Women are classified as having a low risk of GDM if they meet all of the following characteristics: age < 25 years, normal weight before pregnancy, belonging to an ethnic group with a low prevalence of GDM, no known diabetes in first degree relatives, no history of poor obstetric outcome. High risk must be assessed if any of the following risk factors are present: marked obesity (BMI ≥ 30 kg/m^2^), personal history of GDM, family history of Type 2 diabetes in first-degree relatives. Patients with intermediate characteristics have to be defined as at average risk. Screening with an Oral Glucose Challenge Test (OGCT) followed by an Oral Glucose Tolerance Test (OGTT) on that subset of women exceeding the glucose threshold on the OGCT, or a direct OGTT is recommended as soon as feasible in high risk patients. Women of average risk should be tested at 24–28 weeks of gestation, and low risk status requires non glucose testing.

The island of Sardinia, Italy, has an average yearly standardized incidence rate of 33.4 per 100000 (95% C.I. 30.60, 35.88) of Type 1 diabetes, which is the second highest in the world after Finland [19-22], and a high prevalence of other autoimmune diseases Type 1 diabetes related, such as Multiple Sclerosis, Celiac Disease, Autoimmune Thyroid Disease and others [23-26]. For these reasons, Sardinia represents an ideal observatory for investigating genetic and immunological factors. Nevertheless, the prevalence of Type 2 diabetes (3,75% in males e 2,74% in females) in the Sardinian population is similar to that of other, non high-risk populations [27]. We recently reported a very high prevalence (22.3%) of GDM in a large group of Sardinian women [28] compared to that reported in other Italian regions, where it ranges from 2.3 to 10% [29-33]. This high prevalence is only partially explainable by our extended screening procedure, and seems to be in contrast with the reported prevalence of Type 2 diabetes in Sardinia and with the average frequency of GDM in a country known for its high occurrence of Type 1 diabetes, such as Finland [34].

In order to better explain the reasons for such a high prevalence we sought to characterize our obstetric population through the analysis of risk factors, their prevalence and their association with the development of GDM.

## Methods

Screening and diagnosis of GDM were performed according to the American Diabetes Association (ADA) criteria[10] in 1103 pregnant volunteers (247 GDM and 856 control women), consecutively referred to our Obstetric Diagnostic Centre, as previously reported [28]. All subjects were white and of Sardinian descent. None of the participants had Type 2 diabetes outside the index pregnancy, any significant medical problems, nor were they taking any medications known to affect glucose metabolism. The purpose and nature of the study were explained to all subjects, and their consent was obtained before participation. The local Institutional Review Board approved the study.

To avoid undiagnosed cases of GDM, an Oral Glucose Challenge Test (OGCT) was performed at three different gestational ages (16–18 weeks, 24–26 weeks, and 30–32 weeks), and 130 mg/dl was chosen as the glycemic threshold for the Oral Glucose Tolerance Test (OGTT). The ADA recommendations (Carpenter and Coustan criteria: two or more values meeting or exceeding the following: fasting: 95 mg/dl, 1 h: 180 mg/dl, 2 h: 155 mg/dl, 3 h: 140 mg/dl) were used to diagnose GDM.

The risk of GDM was assessed according to the Fourth International Workshop on GDM [1]. Briefly, women were classified as having a low risk of GDM if they met all the following characteristics: age ≤ 25 years, normal weight before pregnancy, belonging to an ethnic group with a low prevalence of GDM, no known diabetes in first degree relatives, no history of poor obstetric outcome. High risk was assessed if even one of the following risk factors was present: marked obesity (BMI ≥ 30 kg/m^2^), personal history of GDM, family history of Type 2 diabetes in first-degree relatives. Patients with intermediate characteristics, such as age ≥ 25 years, Type 2 diabetes in second degree relatives, overweight (BMI: 25–30 kg/m^2^), prior poor obstetrical outcomes usually associated with GDM (macrosomia, polydramnios, repeated abortions, foetal death) were classified as having an "average risk".

### Statistical analysis

Statistical differences between GDM and non-GDM patients were performed with a Student t-test for continuous variables and with a Chi-Square test for others nominal variables. To evaluate association with GDM, risk factors were introduced in a model of logistic regression, using a selection procedure by steps. Classes of risk (high, average, low risk) were analysed with a 2 × 2 contingency table, to validate risk assessment strategy by sensitivity and specificity evaluation. All analysis was performed using SPSS v 12.0 software. For all analysis, *P *< 0.05 was considered to be statistically significant.

## Results

The risk assessment for GDM and risk factors distribution are shown in Table 1. In the overall population, 6.4% (N = 71) were classified as "low risk", 72.6% (N = 801) as "average risk", and 20.9% (N = 231) as "high risk". The main factor responsible for the low prevalence of "low risk" patients was that the majority of patients were ≥ 25 years of age. Factors contributing to a "high risk" classification (verified in 231 women) included Type 2 diabetes in a 1^st ^degree relative (67%), BMI ≥ 30 (23.3%) and history of prior GDM (22%). Multiple risk factors were present in 37.4% of average risk patients and in 11.6% of high-risk patients.

**Table 1:** Risk factor distribution in the overall population and in the two classes of risk

	Overall population (N = 1103) %	Average Risk 72.6% (N = 801) %	High Risk 20.9% (N = 231) %
Age ≥ 25 y	89.3	94. 6	
T2DM in 2^nd^-degree relative(s)	18.5	25.4	
Overweight (25 < BMI < 30)	14.5	19.9	
T2DM in 1^st^-degree relative(s)	14.2	-----	67
Prior poor obstetrical outcomes	12.1	16.7	
Obesity (BMI ≥ 30 kg/m^2^)	4.9	-----	23.3
Prior GDM	4.6	-----	22
One risk factor		62.2	88.3
More than one risk factor		37.4	11.6

The comparison between patients' characteristics in GDM and non-GDM subjects is shown in Table 2. Women diagnosed with GDM had a higher probability of having a first degree relative with Type 2 Diabetes (26.4 vs 10.7%, *P *< 0.001), whereas the presence of Type 2 diabetes in second degree relatives was equally represented in GDM and non-GDM patients. When divided into age groups, only patients over 35 had a higher likelihood of becoming gestational diabetics, i.e., 38.4% of the GDM cases were older than 35 (vs 22.5% of the non-GDM cases, *P *< 0.01), while the age group 25–35 was represented equally in GDM and non-GDM group.

**Table 2:** Comparison between characteristics and risk factors in GDM and non-GDM patients

	GDM N = 247	Non-GDM N= 856	*P *value
High risk [N, (%)]	98 (39.8)	133 (15)	< 0.001
Average risk [N, (%)]	143 (57.8)	658 (76.8)	< 0.001
Low risk [N, (%)]	5 (2.0)	66 (7.7)	< 0.01
Age (mean ± SE)	32.8 ± 0.2	30.5 ± 0.1	< 0.001
Age < 25 [N, (%)]	7 (2.8)	110 (12.8)	< 0.001
Age 25–34 [N, (%)]	145 (58.7)	554 (64.7)	NS
Age ≥ 35 [N, (%)]	95 (38.4)	193 (22.5)	< 0.01
BMI (media ± SE)	23.7 ± 0.2	22.1 ± 0.1	< 0.01
Obesity [N, (%)]	28 (11.3)	26 (3.0)	< 0.001
Overweight [N, (%)]	46 (18.6)	114 (13,3)	< 0.05
Type 2 diabetes in 1st-degree relative(s) [N, (%)]	65 (26.4)	92 (10.7)	< 0.001
Type 2 diabetes in 2nd-degree relative(s) [N, (%)]	46 (18.6)	158 (18.4)	NS
Prior GDM [N, (%)]	27 (10.9)	24 (2.8)	< 0.001
Prior poor obstetrical outcomes [N, (%)]	91 (10.6)	43 (17.5)	< 0.001

P = NS, Not Significant.

A history of prior GDM was also associated with a higher risk of developing GDM in the index pregnancy (10.9% of the GDM cases had a personal history of prior GDM vs 2.8% in non-GDM women, *P *< 0.001). In contrast, prior poor obstetrical outcomes were mostly present in non-GDM patients (17.5% vs 10.6%, respectively, *P *< 0.001).

However, the risk assessment differentiated GDM from non-GDM patients only for the high-risk definition, significantly more frequent in women subsequently diagnosed with GDM (39.8% vs 15%, *P *> 0.001), whereas non-GDM patients belonged more frequently to the average risk category (76.8% vs 57.8%, P < 0.001, Power test: 0.80). The low risk assessment better characterized the subjects who never developed GDM (7.7% vs 2.0%, *P *< 0.01).

These results were substantially confirmed by the logistic regression analysis (Table 3): obesity, strong family history, prior GDM and age were significantly (P < 0.001) associated with GDM, but not prior poor obstetrical outcomes and presence of Type 2 diabetes in second degree relatives, which characterized the definition of average risk. Similarly, when analysed in a contingency table (Table 4), only the high-risk definition was significantly associated with GDM development (Odd Ratio: 3.6, 95% CI: 2.6–4.9), whereas the average risk definition was protective (OR: 0.4, 95% CI: 0.09–0.6), as was the low risk designation (OR: 0.2, 95% CI: 0.3–0.5). Sensitivity, specificity, positive and negative predictive values for all three risk categories are reported in Table 4. The low risk definition showed a sensitivity of 2%, and specificity of 92%, whereas the high risk definition showed a specificity of 84.5%, and a sensitivity of 39.8%.

**Table 3:** Risk factors association with GDM

	OR	CI
Obesity (BMI ≥ 30 kg/m^2^)	3.7	2.08–6.8
Overweight (25 < BMI < 30 kg/m^2^)	1.4	1.00–2.21
Prior GDM	3.1	1.69–5.69
T2DM in 1^st^-degree relative(s)	2.6	1.81–3.86
Age, years	1.08	1.05–1.12

OR, Odds Ratio; CI, Confidence Interval

**Table 4:** Validation of risk assessment strategy

	OR	95% CI	Sensitivity	Specificity	PPV	NPV
High Risk	3.6	2.6–4.9	39.8%	84.5%	42.4%	83.4%
Average Risk	0.4	0.09–0.6	58%	23%	17.9%	66.9%
Low Risk	0.2	0.3–0.5	2%	92%	7%	77%

OR, Odds Ratio; CI, Confidence Interval; PPV, Positive Predictive Value; NPV, Negative Predictive Value

## Discussion

This study was performed in order to verify whether some characteristics of our obstetric population could explain the high prevalence of GDM. Differences in screening programs and diagnostic criteria are the main factors which make it difficult to compare frequencies of GDM among various populations [2,3]. It would have been more correct to compare our prevalence of GDM with that of another Italian region which used the same screening methods, however no such study has been published so far. Nevertheless, although not conclusive, a number of considerations can be made as a result. The extended screening procedure we performed, namely a lower glycemic threshold and three tests performed throughout the index pregnancy, can only partly explain the difference which resulted between other countries and other Italians regions [29-33]. It is well known that lowering the threshold of the OGCT increases sensitivity, at the expense of a slightly lower specificity of the test. By using a lowered threshold of the OGCT, we were able to diagnose an additional 12% of our cases of GDM, similar to findings reported by others [7,35,36], although the prevalence of GDM in their populations ranged from 2 to 6.8%. Thus, the use of a lower threshold does not fully explain such an elevated prevalence. Another important consideration to be made is that almost half our GDM cases were diagnosed at 30–32 weeks of gestation, which goes along with the progressively increasing insulin resistance during pregnancy [37,38]. Therefore, it is possible that other studies, where screening was performed at 24–28 weeks, may have missed a significant number of the cases of GDM. On the other hand, analyzing only the diagnoses made through early and mid-pregnancy screening, our prevalence is still surprisingly high (12%).

Our study showed that the "average risk" definition, which characterized the majority of our patients, does not identify patients at risk of GDM. This also excludes the possibility of a selection bias to invalidate our results (that is, too many "at risk" patients). On the contrary, despite the low sensitivity of the high-risk definition, the high odd ratio and good specificity seem to appropriately characterize the population really "at risk" of GDM. Similarly, the low risk definition identifies women who are unlikely to develop GDM.

The analysis of the individual prevalences of risk factors does not seem to show an unusual prevalence of any of the risk factors sufficient to justify the high prevalence of GDM. We compared our results from the general obstetric population with those reported in other populations [3,9,17,30,39]: in another Italian region, in the USA and in northern and southern Europe (Table 5).

**Table 5:** Comparison of GDM and risk factors prevalence with other studies

	Present study Sardinia, Italy	Di Cianni Tuscany, Italy ^(30)^	Danilenko-Dixon USA ^(9)^	Jimenez-Moleon, Spain ^(3)^	Ostlund, Sweden ^(40)^
GDM prevalence	22.3%	8.74%	3%	2.5%	1.7%
Obesity (BMI ≥ 30 kg/m^2^)	4.9%	4.7%	NA*	5.2%	7.9%
Overweight (25 < BMI < 30 kg/m^2^)	14.5%	12.6%	NA	NA^§^	NA ^##^
Prior GDM	4.6%	NA	2.1%	0.8%	1.3%
T2DM in 1^st^-degree relative(s)	14.2%	18.1% **	7.7%**	14.8%**	9.4%**
Age ≥ 25 yrs	89.3%	NA	82.2%	80.3%	73.6
Prior poor obstetrical outcomes	12.1%	NA	NA***	NA ^#^	NA^§§^

NA = Not available

* In this study normal body weight is defined as BMI of < 27 kg/m^2^. The  percent of women with BMI > 25 was 33%, and the one of BMI > 27% was 22%.

^§ ^In this study patients with BMI 27–30 (7.1%) were evaluated

^## ^In this study BMI ≥ 25 (28.3%) and ≥ 28 (12.3%) were evaluated

** In this study it is not specified if familiarity is for Type 1 or Type 2 diabetes

***In this study only prior macrosomia (14.5%) and prior foetal death (0.5%) were evaluated

^# ^In this study prior macrosomia (4.9%), prior fetal death (1%), and congenital malformation were evaluated. The latter were not evaluated in our study (0.4%), whereas repeated abortions have not been reported.

^§§ ^In this study only prior macrosomia was evaluated

The analysis is rendered difficult by the often different definitions of the individual factors, for example obesity, over-weight or poor obstetrical outcome, and in many cases a comparison with our study was not possible.

Obesity is a well known risk factor for GDM, which is strongly associated with GDM in our patients (OR: 3.7), but it was present in only 4.9% of our overall population, similarly to what has been found elsewhere in Italy [30] and Spain [3], but considerably lower than the prevalence reported (7.9%) in Sweden [39], and probably in the USA, where 22% of patients have been reported to have a BMI > 27. By contrast, in both the USA and Sweden, GDM prevalence is very low. Thus, it seems unlikely that the high prevalence of GDM in our population can be explained by our prevalence of obesity.

The prevalence of prior GDM is higher to that reported in other countries. This prevalence is obviously influenced by the different proportion of primiparas and multiparas in patient groups, not evaluated in our study. The Swedish study [39] reported a percent of 1.3% in primiparas and 2.3% in multiparas, which is half the prior GDM in our patients. Thus, it seems more likely that the prevalence of prior GDM in our patients is linked to our GDM prevalence.

The results on family history vary considerably in the studies evaluated, since the prevalence in our population is similar only to that reported in Spain, lower to that reported elsewhere in Italy and higher to that reported in the USA and Sweden. However, several factors can influence this parameter. Firstly, none of the studies examined specify whether the familiarity evaluated was for Type 1 or Type 2 diabetes. Furthermore, it can be difficult to obtain an accurate medical family history, especially in patients from lower social status groups. This factor is also influenced by age because if the patient is young Type 2 diabetes is not yet detectable in parents. Thus, although in absolute terms the prevalence of this factor in our general population does not seem elevated, nothing can be concluded from a comparison with other countries.

Although age is the most common risk factor, the percentage of women over 25 (89%), was only slightly higher than that reported in the others studies, ranging from 82.2% in the USA [9] to 73.6% in Sweden [39] and is association with GDM, though significant, is weak (OR:1.08). Furthermore, only women over 34 (35 yrs or more) were more represented in our GDM group. This could mean that, at least in our population, age is a risk factor only if women are over 34. This group of women is 26% of the overall population, very close to the 24.6% reported in other Italian regions with a lower GDM prevalence [30]. Hence, the older age of our patients does not seem sufficient to explain such a high GDM prevalence.

Furthermore, none of the studies which were compared with our findings had examined prior obstetrical outcomes, thus a comparison between prevalences was impossible. However, this factor did not show a significant association with GDM in our population, in agreement with the findings of Kautzky-Willer [17], where the same parameters as ours were evaluated (macrosomia, polydramnios, repeated abortions, fetal death).

## Conclusion

Such a high prevalence of GDM in our population does not seem to be related to the abnormal presence of some known risk factors, and appears in contrast with the prevalence of Type 2 diabetes.

Since Sardinia is a privileged territory with a very peculiar genetic subset, which links Type 1 diabetes and other autoimmune diseases to this Island, further studies to examine the genetic and immunological pool of Sardinian women of childbearing potential are needed.

## Competing interests

The authors declare that they have no competing interests.

## Authors' contributions

CM conceived of the study, its design and coordination and draft the manuscript, RB participated in the design of the study and helped to draft the manuscript, LM performed the statistical analysis, SS, MM, EP, NG and PZ participated in patients recruitment and data collecting, GBM made substantial contribution to conception and design and was ultimately responsible for this work. All authors read and approved the final manuscript.
